# Supplementary material for: Live cell imaging of single RNA molecules with fluorogenic Mango II arrays
Source: Nat Commun. 2020 Mar 9;11:1283. doi: 10.1038/s41467-020-14932-7 (PMC7062757; doi:10.1038/s41467-020-14932-7)
Supplement: Supplementary file 1 — Supplementary Information [file 41467_2020_14932_MOESM1_ESM.pdf]

Supplementary Information

Live Cell Imaging of Single RNA Molecules with Fluorogenic Mango II Arrays

Cawte *et al.*

Supplementary Table 1. DNA constructs, primers and FISH probes

Constructs, primers and probes	Sequence (5' → 3')
Mango II	GGCACGTACG AAGGAGAGGA GAGGAAGAGG AGAGTACGTG C
M2 x3 array (5 nt)	GGCACGTACG AAGGAGAGGA GAGGAAGAGG AGAGTACGTG CTTTTTGGAC ATGCCGAAGG AGAGGAGAGG AAGAGGAGAG GCATGTCCTT TTTGGGTGCA TCGAAGGAGA GGAGAGGAAG AGGAGAGATG CACCC
M2 x3 array (1 nt)	GGCACGTACG AAGGAGAGGA GAGGAAGAGG AGAGTACGTG CTGGACATGC CGAAGGAGAG GAGAGGAAGA GGAGAGGCAT GTCCTGGGTG CATCGAAGGA GAGGAGAGGA AGAGGAGAGA TGCACCC
Broccoli	GGAGACGGTC GGGTCCAGAT ATTCGTATCT GTCGAGTAGA GTGTGGGCTC C
Broccoli Double (10 nt)	GGAGACGGTC GGGTCCAGAT ATTCGTATCT GTCGAGTAGA GTGTGGGCTC CCTCTCTCTC TGGAGACGGT CGGGTCCCTC GATTCGTCGA GGTCGAGTAG AGTGTGGGCT CC
Broccoli Triple (5 nt)	GGAGACGGTC GGGTCCAGAT ATTCGTATCT GTCGAGTAGA GTGTGGGCTC CTTTTTGGCT ACGGTCGGGT CCCTCGATTC GTCGAGGTCG AGTAGAGTGT GGGAGCCTTT TTGGTCACGG TCGGGTCCTC TAATTCGTTA GAGTCGAGTA GAGTGTGGGG ACC
5’ AgeI mCherry	AGC ACC GGT CGC CAC CAT GGT GAG
3’ BamHI SV40 pA	CCC GGA TCC TTG TTG TTA ACT TGT TTA TTG
pmax_M2/MS2_Bam-Pvu QuickChange_1	ATA AGT ACC GTA CGA TCG TTA TCA CTC GAG CCT GGA ATA C
pmax_M2/MS2_Bam-Pvu QuikChange_2	GTA TTC CAG GCT CGA GTG ATA ACG ATC GTA CGG TAC TTA T
5' XbaI M2 array	AGC TCT AGA CGA TCG TAC GGT ACT TAT TGC CAA GAA AGG
3' BclI SV40 pA	AAC TGA TCA TTG TTG TTA ACT TGT TTA TTG
5’ BamHI B-act 3’UTR	TAG GGA TCC GCG GAC TGT TAC TGA GCT G
3’ BclI B-act 3’UTR	TTA TGA TCA TAG GAG TGG GGG TGG C
5’ Halo Bact insert Gibson	GGA GAA TTG GCT AGC ACC GGT CCG GCC GCC ACC ATG GAT TAC
3’Halo Bact insert Gibson	CTT GGC AAT AAG TAC CGT ACG ATC GTA GGA GTG GGG GTG GCT TTT G

3' Halo Bact Δ3UTR insert Gibson	CTT GGC AAT AAG TAC CGT ACG ATC GCT AGC TAG CCT AGA AGC ATT TGC G
pLenti-M2 array Gibson_Fwd	CGA TCG TAC GGT ACT TAT TGC CAA G
pLenti-M2 array Gibson_Rev	GAC CGG TGC TAG CCA ATT CTC C
M2-AF488 smFISH_1	AF488N//CGGCATGTCC AAAAAGCACG
M2-AF488 smFISH_2	AF488N//ATGCACCCAA AAAGGACTG
M2-AF488 smFISH_3	AF488N//TACGTGCCAA AAAGGGTGCA
MS2v5-FAM smFISH_1	FAM//TGATTGTGAAGTGTCGGGTG
MS2v5-FAM smFISH_2	FAM//TCCACCCTTGTGTATTGTAC
MS2v5-FAM smFISH_3	FAM//TGTAATGTGTCTGGAGGGTG
MS2v5-FAM smFISH_4	FAM//GCTTCTGTTTGATTGGATTT
MS2v5-FAM smFISH_5	FAM//GATGGTGATTCCTTGTTGTA
MS2v5-FAM smFISH_6	FAM//GTATATTGCACAGGGAATCC
MS2v5-FAM smFISH_7	FAM//GATATTCGGGAGGCGTGATC
MS2v5-FAM smFISH_8	FAM//ACGCACTGAATTCGAAAGCC
MS2v5-FAM smFISH_9	FAM//ATTCGACTCTGATTGGCTGC
MS2v5-FAM smFISH_10	FAM//CTCTTCGCGAAAGTCGACTT
MS2v5-FAM smFISH_11	FAM//TAAGAATGGCGCGAAGGCTG
MS2v5-FAM smFISH_12	FAM//GTAGGGGAGAGTGTGGTTTG
MS2v5-FAM smFISH_13	FAM//CAGGAACGCTGATGCTGTTC
MS2v5-FAM smFISH_14	FAM//TTTTCTTGAGTTGGGTACTG

MS2v5-FAM smFISH_15	FAM//TGATGCTGCATGGGGACATA
MS2v5-FAM smFISH_16	FAM//TTGGGGATGTATTCTTGGGG
MS2v5-FAM smFISH_17	FAM//TTGGTGCTCGGATGTGATTT
MS2v5-FAM smFISH_18	FAM//AAGAAACAACACTCCGAGCC
MS2v5-FAM smFISH_19	FAM//ATGGAGGGTTTGTCCAGTTG
MS2v5-FAM smFISH_20	FAM//TTTGTCTTGTTGGTGAGAGT
MS2v5-FAM smFISH_21	FAM//CTGATGCTGCTTCGAGAAGA
MS2v5-FAM smFISH_22	FAM//GTATGCTCGAGTGTTTCGAA
MS2v5-FAM smFISH_23	FAM//GATCGTCCACCCAAGAAATA
MS2v5-FAM smFISH_24	FAM//AATTCGTGAGAGCATGGGTG
MS2v5-FAM smFISH_25	FAM//TCGTATTGGACGTGGAACGA
MS2v5-FAM smFISH_26	FAM//TCGTGATCCCGAAAGGTAAG
MS2v5-FAM smFISH_27	FAM//ATCGTGCATGCTTGAATGTC
MS2v5-FAM smFISH_28	FAM//GTTGAGACTTGTGGAGCATG
MS2v5-FAM smFISH_29	FAM//TGAACCCATTTGGTAGTTTC
MS2v5-FAM smFISH_30	FAM//TTTGAGGTAGGAGTGGGTTC
MS2v5-FAM smFISH_31	FAM//TTGCCAGTTTTGTGGGAAGA
MS2v5-FAM smFISH_32	FAM//TTTGGTATGTTGGAATGGGC
MS2v5-FAM smFISH_33	FAM//GATGCTGTACCAGTAATTGT
MS2v5-FAM smFISH_34	FAM//TAGTAGTGAGAGATGTGGGC

MS2v5-FAM smFISH_35	FAM//TGCTGAACGGTTTGGTTTTT
MS2v5-FAM smFISH_36	FAM//TTGATTTTTCCGTGTGTACC
MS2v5-FAM smFISH_37	FAM//GTCTTTCGTATTTGTAAACC
MS2v5-FAM smFISH_38	FAM//TTGCGCTGGACGAAAGCGTG
MS2v5-FAM smFISH_39	FAM//CCGTCGGATGTTTTTCGTAA
MS2v5-FAM smFISH_40	FAM//GGTTGTAAGTTTGTGGGTTG
MS2v5-FAM smFISH_41	FAM//TTGATGTACGGTGCGGTGAT
MS2v5-FAM smFISH_42	FAM//ATCGATATGAGATCTGAGGT

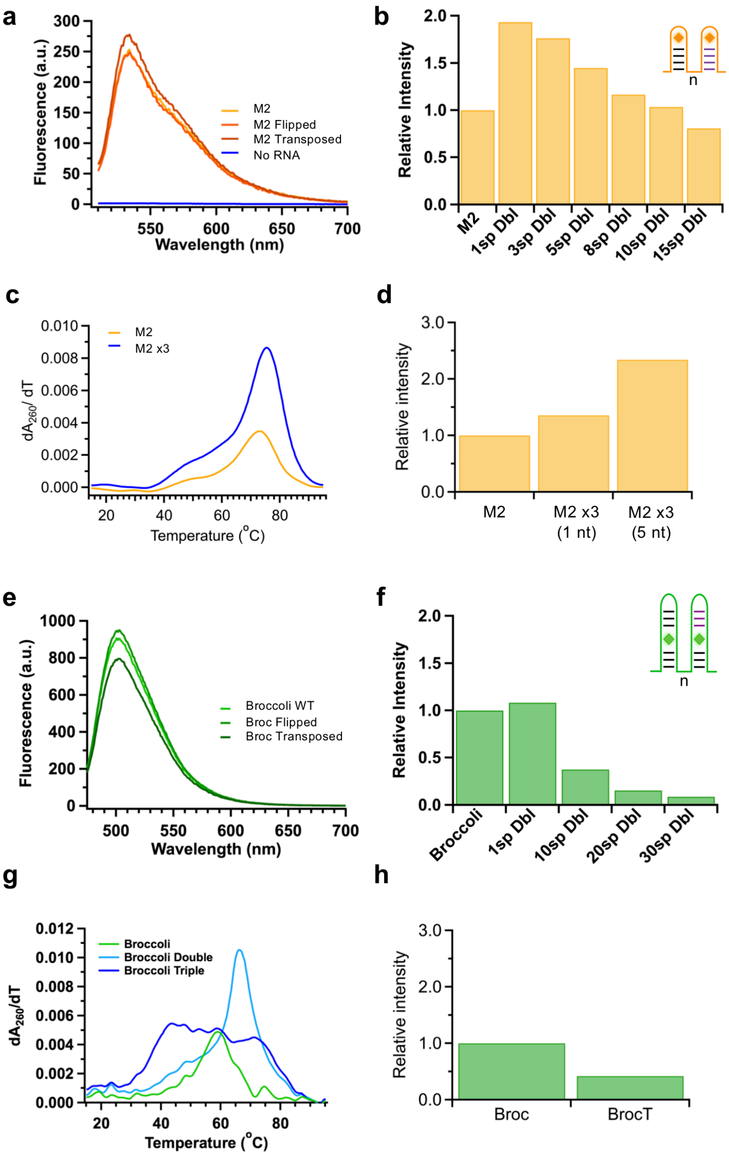

Supplementary Figure 1 | *In vitro* characterisation of aptamer arrays

(**a**) Fluorescent spectra of individual mutant Mango sequences (40 nM) either flipped or transposed in the presence of TO1-Biotin (200 nM). (**b**) Relative fluorescent intensity of dimeric Mango constructs with alteration of the nucleotide spacing (n) between adjacent aptamers. (**c**) Differentials of the UV melting curves for Mango II and the Mango II x3 (5nt) array. Adapted from main figure 1 to aid comparison with Broccoli constructs. (**d**) Relative fluorescent intensity of Mango II and the Mango II x3 (1 nt) and (5nt) arrays. (**e**) Fluorescent spectra of individual mutant Broccoli sequences (40 nM) with the P3 stem either flipped or transposed in the presence of DFHBI (1 μM). (**f**) Relative fluorescent intensity of dimeric Broccoli constructs with alteration of the nucleotide spacing (n) between adjacent aptamers. (**g**) Differentials of the UV melting curves for Broccoli the Broccoli (10 nt) dimer and the Broccoli trimeric array. (**h**) Relative fluorescent intensity of Broccoli and the Broccoli trimeric array (BrocT).

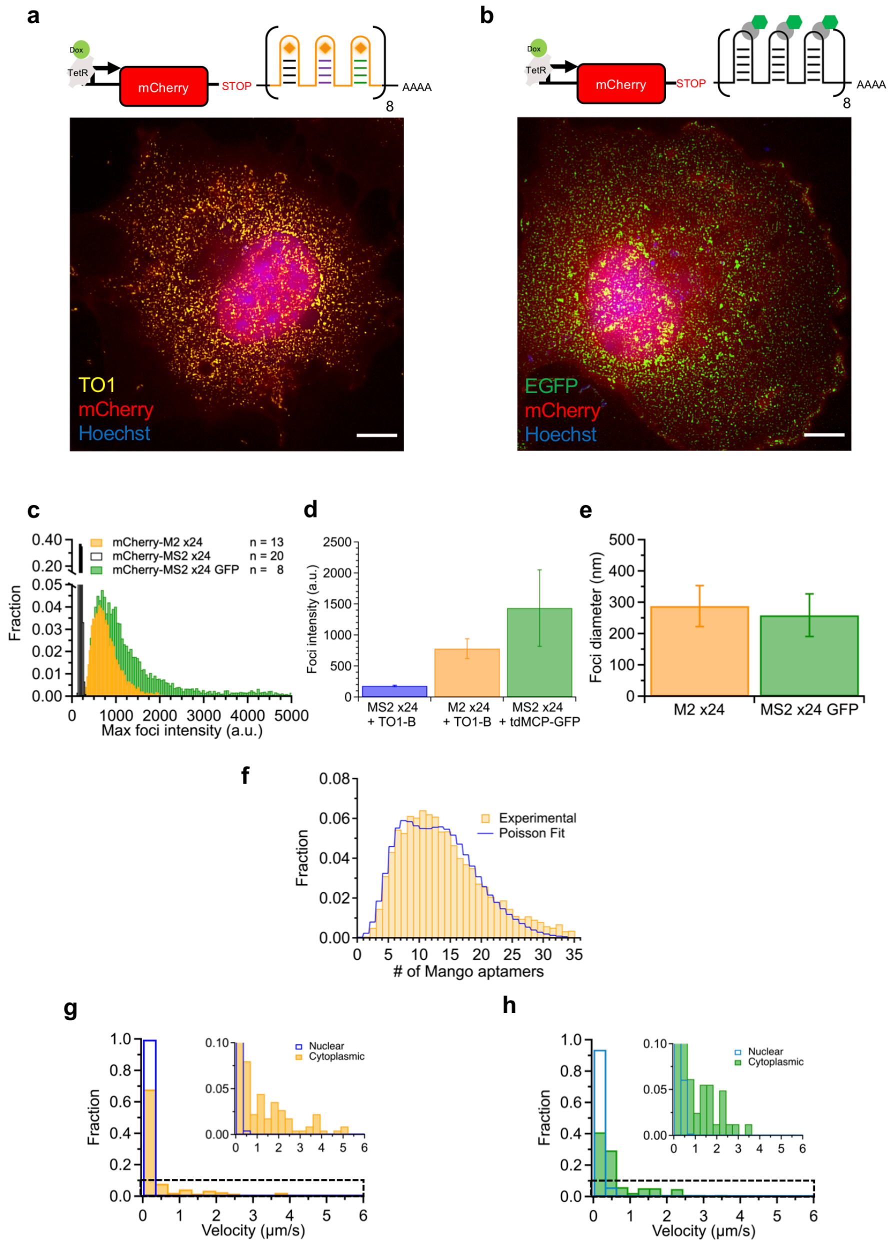

Supplementary Figure 2 | Comparison of M2 x24 and MS2v5 x24 labelled single RNA molecules

(**a**) Schematic of the Dox inducible mCherry-M2 x24 construct and a representative maximum projection of a cell displaying evenly dispersed single molecules. Cells stained with 200 nM TO1-B. (**b**) Schematic of the Dox inducible mCherry-MS2v5x24-tdMCP-EGFP construct and a representative maximum projection of a cell displaying evenly dispersed single molecules. Both scale bars = 10 µm, in Cos-7 Cells. (**c**) Distribution of single molecule intensities for cells expressing the mCherry-M2 x24 construct + TO1-B (200 nM - yellow), mCherry-MS2v5 x24 + TO1-B (200 nM - black) and mCherry-MS2v5 x24-tdMCP-EGFP (green), 7008, 30,429 and 10,338 foci respectively. N shown in figure depicts number of cells used for analysis. Figure adapted from main figure 2f for direct comparison with EGFP foci. (**d**) Mean foci intensity of

each construct with the error shown as standard deviation. (**e**) Mean foci diameter of M2 x24 and MS2v5 x24-tdMCP-EGFP RNAs with the error shown as standard deviation. (**f**). Foci intensity distribution shown as # of Mangos as approximated using background subtraction of 150 a.u. and division of the calculated mean step size of 40 a.u (**Fig. 2e**). Following normalisation, the distribution was fit with three Poisson distribution fit as described in the materials and methods. (**g**) Distribution of nuclear (blue) and cytoplasmic (orange) single molecule velocities for the mCherry-M2 x24 construct, n = 229 and 224 respectively. (**h**) Distribution of nuclear (light blue) and cytoplasmic (green) single molecule velocities for the phage-CMV-CFP-MS2SLx24-tdMCP-EGFP construct, n = 461 and 163 respectively.

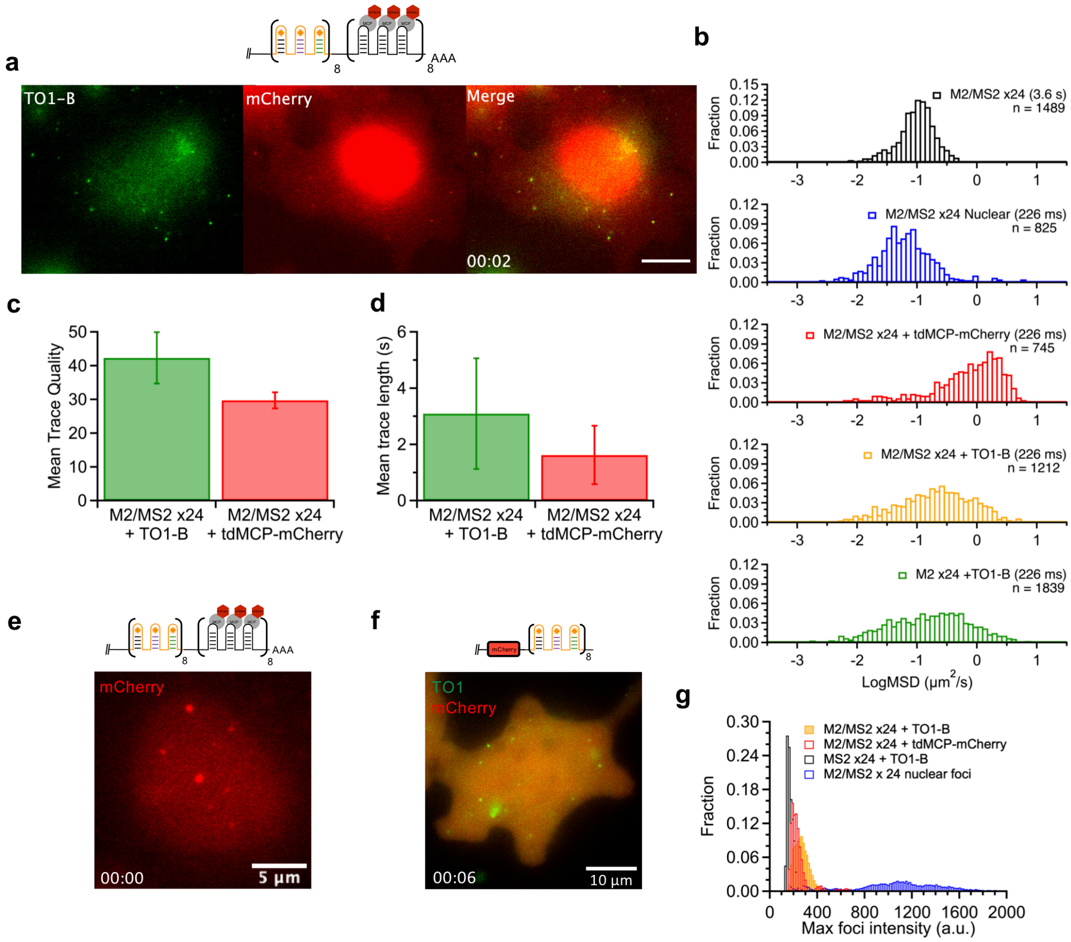

Supplementary Figure 3 | Live-cell imaging of M2 x24 and MS2-SLx24-tdMCP-mCherry (**a**) Live-cell snapshot of a cell expressing the M2/MS2-SLx24-tdMCP-mCherry construct depicting colocalization of the two signals. TO1-B channel – green, mCherry channel – red, scale bar = 10 μm and time shown in seconds. Still taken from Supplementary Movie 4. (**b**) Distributions of LogMSD for live cell trajectories for M2/MS2-SLx24 tdMCP-mCherry at 3.6 s frame rate (black), nuclear M2/MS2-SLx24 tdMCP-mCherry foci at 226 ms frame rate (blue), cytosolic M2/MS2-SLx24 tdMCP-mCherry foci at 226 ms frame rate (red), M2/MS2-SLx24 + TO1-B at 226 ms frame rate (yellow) and mCherry-M2 x24 + TO1-B at 226 ms frame rate (green). N shown as the number of trajectories analysed from a total of 26 cells. (**c**) Mean trace quality as determined from the TrackMate plugin for M2/MS2-SLx24 RNA in the presence of TO1-B and tdMCP-mCherry. Green bar represents 1212 traces analysed in the TO1-B channel (green). Red bar represents 745 traces analysed in the mCherry channel (red). (**d**) Mean trace length as determined from the TrackMate plugin for M2/MS2-SLx24 RNA in the presence of TO1-B and tdMCP-mCherry. Green bar represents 1212 traces analysed in the TO1-B channel (green). Red bar represents 745 traces analysed in the mCherry channel (red). Error bars depict standard deviation of the data sets. (**e**) Representative image of M2/MS2-SLx24 + tdMCP-mCherry nuclear foci observed in the red channel, scale bar = 5 μm

and time shown in seconds. Still taken from Supplementary Movie 7. (**f**) Representative snapshot of mCherry-M2 x24 foci in live cell showing presence of foci fluorescing specifically in the green channel and mCherry signal in the red channel, scale bar = 10 μm and time shown in seconds. Still taken from Supplementary Movie 1. (**g**) Live-cell foci intensity distributions for mCherry-MS2v5 x24 + TO1-B (black), M2/MS2-SLx24 + TO1-B (yellow), cytosolic M2/MS2-SLx24 + tdMCP-mCherry (red) and nuclear M2/MS2-SLx24 + tdMCP-mCherry (blue). n = 33,370, 17,544, 5881 and 12, 937 foci from a total of 26 cells. Data adapted from main figure 3 h for comparison with nuclear foci distribution.

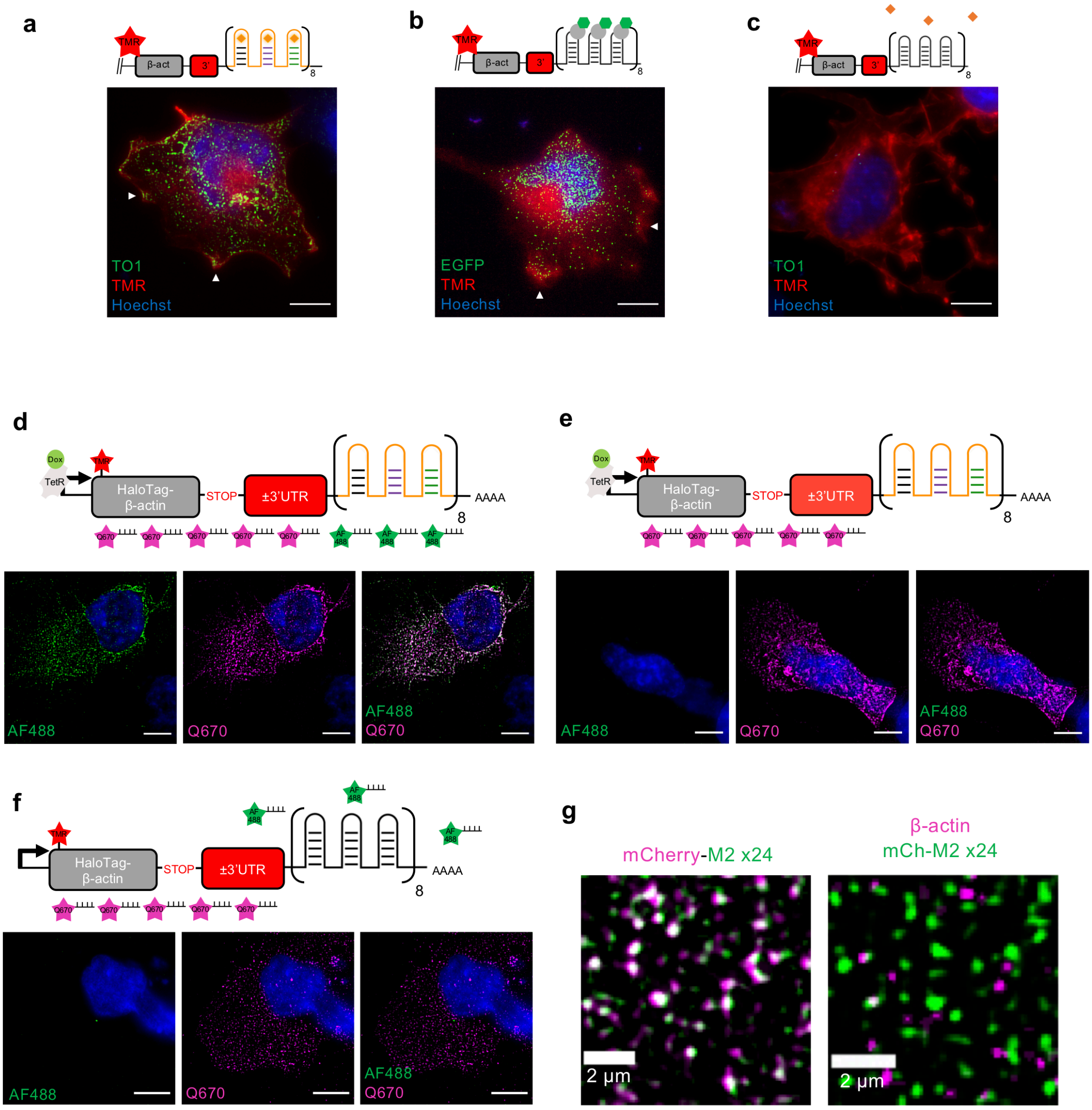

Supplementary Figure 4 | Validation of β-actin mRNA constructs

(**a - c**) Expression of Halo-tagged β-actin mRNA constructs in Cos-7 cells labelled with TMR-Halo ligand showing local expression of β-actin protein. (a) depicts Halo-β-actin-3'UTR M2 x24 + TO1-B expression, (b) Halo-β-actin-3'UTR MS2v5 x24 + tdMCP-EGFP and (c) Halo-β-actin-3'UTR MS2v5 x24 + TO1-B. White arrows depict mRNA signal at cell periphery, mRNA signal – green, protein signal – red, nuclear signal – blue and scale bars = 10 μm. (**d**) Diagram and images of Halo-β-actin-3'UTR M2 x24 expression dually labelled with β-actin-Q670 (magenta) and M2-AF488 (green) probes. (**e**) Diagram and images of Halo-β-actin-3’UTR M2 x24 expression singly labelled with β-actin-Q670 (magenta) probes. (**f**) Diagram and images of Halo-β-actin-3'UTR MS2v5 x24 expression dually labelled with β-actin-Q670 (magenta) and M2-AF488 (green) probes. Scale bars = 10 μm. (**g**) Images of mCherry-M2 x24 expression

dually labelled with mCherry-Q670 (magenta) and M2-AF488 (green) probes, left and dually labelled with β-actin-Q670 (magenta) and M2-AF488 (green) probes, right. Scale bars = 2 μm.

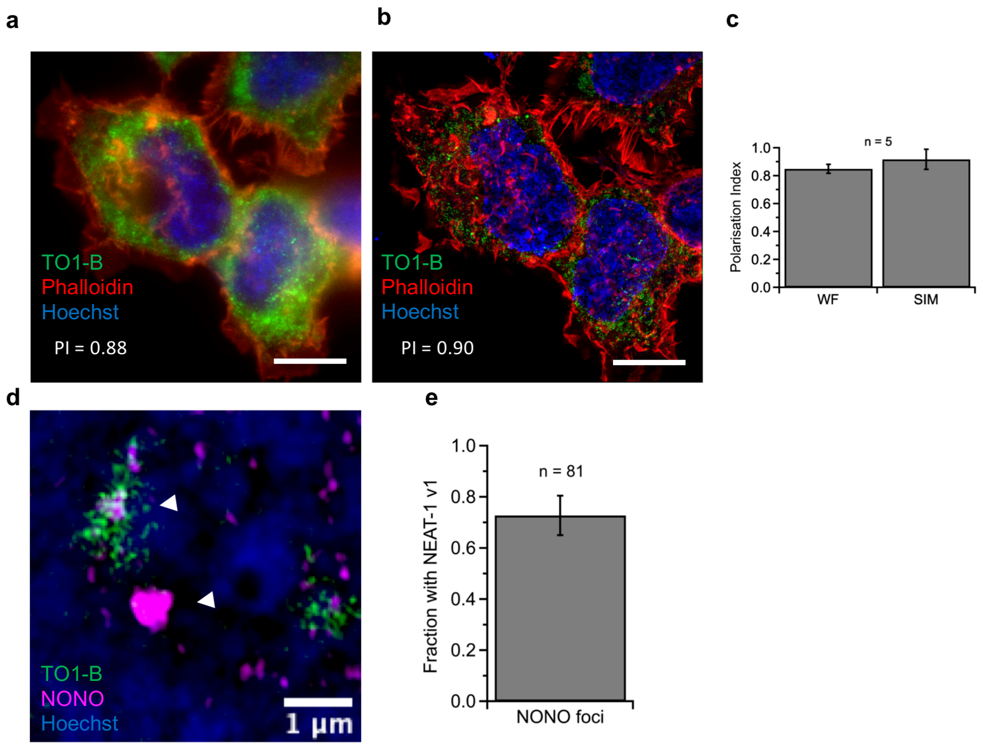

Supplementary Figure 5 | Quantification and validation of SIM images

(**a**) Widefield image of β-actin-Δ3'UTR-M2 x24 + TO1-B with average PI value depicted in the bottom left hand corner (**b**) Structured illumination microscopy image of β-actin-Δ3'UTR-M2 x24 + TO1-B cell with the average PI also depicted in the bottom left corner. Data adapted from main figure 5 for direct comparison of PI values, scale bars = 10 µm. (**c**) Quantification of the average PI value for 5 cells using both widefield and SIM images showing only a minimal change in PI. (**d**) Image of paraspeckle formation either containing NEAT-1 v1-M2 x24 (top left foci) or showing its absence (bottom left foci) as depicted with white arrows. NONO antibody stain (magenta), TO1-B (green) and Hoechst (blue). Scale bar = 1 µm. (**e**) Quantification of 81 paraspeckles showing an association of NEAT-1 v1-M2 x24 with ~ 70% of foci, error bars depicted as standard deviation.
