# Supplementary material for: Live cell imaging of single RNA molecules with fluorogenic Mango II arrays
Source: Nat Commun. 2020 Mar 9;11:1283. doi: 10.1038/s41467-020-14932-7 (PMC7062757; doi:10.1038/s41467-020-14932-7)
Supplement: Supplementary file 3 — Description of Additional Supplementary Files [file 41467_2020_14932_MOESM3_ESM.pdf]

Description of Additional Supplementary Files

File name: Supplementary Movie 1

Description: Expression of the mCherry-M2 x24 construct in Cos-7 cells in the presence of 200 nM TO1-Biotin and imaged at 226 ms per frame in both the green (TO1-B) and red (mCherry) channels as described in the materials and methods. Time in min:sec format and scale bar = 10 µm.

File name: Supplementary Movie 2

Description: Expression of the mCherry-MS2v5 x24 control construct in Cos-7 cells in the presence of 200 nM TO1-Biotin and imaged at 226 ms per frame in both the green (TO1-B) and red (mCherry) channels as described in the materials and methods. Time in min:sec format and scale bars = 10 µm.

File name: Supplementary Movie 3

Description: Expression of the pHAGE-CMV-CFP-MS2-SLx24 construct in HEK293T cell in the presence of tdMCP-EGFP and imaged at 2 s per frame in the green (EGFP). Time in min:sec format and scale bars = 10 µm.

File name: Supplementary Movie 4

Description: Expression of the M2/MS2 SL x24 construct in Cos-7 cells in the presence of 200 nM TO1-Biotin and NLS-tdMCP-mCherry. Cells imaged at 226 ms per frame in both the green (TO1-B) and red (mCherry) channels as described in the materials and methods. Time in min:sec format and scale bar = 10 µm.

File name: Supplementary Movie 5

Description: Tracking of M2/MS2 SL x24 foci in Cos-7 cells in the presence of 200 nM TO1-Biotin and NLS-tdMCP-mCherry. Cells imaged at 226 ms per frame in both the green (TO1-B) and red (mCherry) channels as described in the materials and methods. Time in min:sec format and scale bar = 2 µm, blue trace represents track as computed using the TrackMate plugin.

File name: Supplementary Movie 6

Description: Depiction of both nuclear and cytosolic M2/MS2 SL x24 transcripts in Cos-7 cells in the presence of 200 nM TO1-Biotin and NLS-tdMCP-mCherry. Cells imaged at 226 ms per frame in both the green (TO1-B) and red (mCherry) channels as described in the materials and methods. mCherry channel with enhanced contrast by ~4x in the middle right panel to show presence of low intensity cytosolic single molecules. These are not able to be visualised in the mCherry channel at low contrast settings (middle left panel). Time in min:sec format and scale bar = 10 µm.

File name: Supplementary Movie 7

Description: Visualisation of bright nuclear M2/MS2 SL x24 foci in Cos-7 cells imaged using the NLS-tdMCP-mCherry signal. Cells imaged at 226 ms per frame. Time in min:sec format and scale bar = 5 µm.
